# Inflammation but Not Endothelial Dysfunction Is Associated with the Severity of Coronary Artery Disease in Dyslipidemic Subjects

**DOI:** 10.1155/2009/469169

**Published:** 2009-06-23

**Authors:** Christian F. Rueda-Clausen, Patricio López-Jaramillo, Carlos Luengas, Maria del Pilar Oubiña, Victoria Cachofeiro, Vicente Lahera

**Affiliations:** ^1^Instituto de Investigaciones, Fundación Cardiovascular de Colombia, Floridablanca, Colombia; ^2^Departamento de Fisiología, Facultad de Medicina, Universidad Complutense, 28040 Madrid, Spain; ^3^Facultad de Medicina, Universidad de Santander UDES, Bucaramanga, Colombia

## Abstract

*Introduction*. Endothelial dysfunction and inflammation play a key role in the development of atherosclerosis. The present study evaluated endothelial function, inflammatory parameters, and carotid intima-media thickness (IMT) in dyslipidemic patients with or without coronary artery disease (CAD). *Methods*. Metabolic profile and inflammatory parameters were determined in dyslipidemic patients with (+CAD, *n* = 33) and without (−CAD, *n* = 69) symptomatic CAD. Endothelial function was evaluated by flow mediated dilatation (FMD) and plasma concentration of nitrites and nitrates. Carotid IMT was measured by ultrasound. *Results*. No significant differences were observed in anthropometric hemodynamic or metabolic parameters between the groups. After adjusting by age and medication usage, some inflammatory markers were significantly higher in +CAD; however no significant differences in FMD or plasma levels of nitrites were observed. *Conclusions*. In subjects with dyslipidemia, the presence of CAD is associated with an elevation of certain inflammatory markers and carotid IMT but not with further endothelial dysfunction.

## 1. Introduction

Dyslipidemia together with hypertension and diabetes is major modifiable risk factor for atherosclerotic disease and the subsequent development of cardiovascular events [1–4]. Endothelial dysfunction, which is a condition that has been strongly associated with dyslipidemia, plays a key role in the development and progression of atherosclerosis [5], and it is known to be an independent predictor for cardiovascular events [6, 7]. The reduced availability of nitric oxide (NO) resulting from both a decreased synthesis and/or an enhanced degradation by reactive oxygen species seems to be the major cause of endothelial dysfunction documented in subjects with cardiovascular risk factors including dyslipidemia [8]. It is also well accepted that atherosclerosis can be considered a chronic vascular inflammatory disease [9]. Inflammatory cytokines are responsible for activation of endothelial cells, a condition characterized by the expression of endothelial cell-surface adhesion molecules such as vascular cell adhesion molecule-1 (sVCAM-1) and p-Selectin, that favor the attachment of circulating monocytes to the endothelium [10] and their migration and differentiation in the vascular intima-media layer [11]. The consequence of this persistent migration and cellular differentiation in the subendothelial vascular layers causes an increase in the arterial intima-media thickness, which is considered a highly sensitive marker of atherosclerosis progression [6, 12]. Similarly, C-reactive protein (CRP), which is a well-described inflammatory marker, has been shown to be an independent predictors of future cardiovascular events in both high-risk and healthy subjects [13–15]. Moreover, increased circulating cytokines including tumor necrosis factor alpha (TNF*α*), interleukin-1 beta (IL-1*β*), and interleukin-6 (IL-6) have also been associated with cardiovascular events [16]. However, it remains unclear whether the evaluation of endothelial function and inflammatory markers reflects the same stage in the progression and severity of atherosclerotic disease. A question that is particularly relevant for populations from developing countries who are know to be more susceptible to develop proinflammatory states and insulin resistance [17]. In order to clarify these aspects, the present paper aimed to evaluate endothelial function, plasma levels of inflammatory markers, and carotid intima-media thickness (IMT) as well as the changes in these parameters associated with the presence of clinically documented coronary artery disease (CAD) in dyslipidemic patients.

## 2. Methods

This study included 102 dyslipidemic (LDL Cholesterol >3.33 mmol/L) male patients (25 to 77 years of age) distributed in two groups: subjects without history of cardiovascular events or clinical symptoms of coronary disease (−CAD, *n* = 69) and patients with clinically diagnosed CAD (+CAD, *n* = 33). The clinical criteria for diagnosis of CAD included history of acute myocardial infarction (*n* = 12), coronary artery bypass grafting (*n* = 13), coronary hearth disease diagnosed by arteriography (*n* = 6), and chronic stable angina (*n* = 2) diagnosed at least 6 months previous to the evaluation. Exclusion criteria included: body mass index >35, history of secondary or familiar hypercholesterolemia, diabetes mellitus, abnormal liver function, renal impairment, clinical heart failure (NYHA classes III-IV), clinical vascular events (transitory ischemic accident, peripheral arteries occlusion, or mesenteric artery occlusion) during the last six months, chronic inflammatory diseases, and acute illness or major trauma in the last eight weeks. Due to the well-described effects of lipid lowering medications on endothelial function and inflammatory markers [18–20], and in order to avoid the potential confusion in the data analysis and interpretation, only those subjects who were not receiving this kind of medication at least 3 months previous to the evaluation were included in the study. Given the known variability in the determinations of endothelial function and inflammatory markers among different laboratories, and in order to have a reference point to identify the “normal values” of these parameters, a group of 25 healthy young volunteers was also studied.

A complete medical examination that included cardiovascular risk factor evaluation, vital signs, and anthropometrical measurements (following the Anthropometry Procedures Manual NHANES-2002 [21]) was performed on every subject. Fasting venous blood samples were taken for determination of glucose, creatinine, total cholesterol (TC), HDL cholesterol (HDLc), LDL cholesterol (LDLc), and triglycerides (TG), using standard techniques. Plasma concentrations of IL-6, IL-1*β*, TNF*α*, and soluble fractions of sVCAM-1 and p-Selectin, were measured with quantitative sandwich enzyme immunoassay techniques (R&D Systems, Minneapolis, Minn, USA) as previously described [18]. Ultrasensitive CRP plasma levels were measured with a highly sensitive latex-based turbidimetric immunoassay on a Hitachi analyzer (Sigma Chemical Co, St. Louis, Mo, USA). The concentration of nitrites and nitrates, the stable metabolites of NO [22], was determined in plasma samples obtained after 24 hours of nitrate free diet, using a commercial kit (R&D Systems) that involved the conversion of nitrates to nitrites by the enzyme nitrate reductase. 

Flow mediated dilation (FMD) assessment was performed according to the recommendations of the “International Brachial Artery Reactivity Task Force” [23]. This technique was validated by our group in a Colombian population and published elsewhere [24, 25]. All measures were performed in a temperature-controlled room (24°C), in the morning, with a fasting period of at least 10 hours in all the subjects. Brachial artery diameter and blood flow velocity were imaged using a 7.5-MHz linear-array transducer ultrasound system (Aloka, Vario view SDD 2200, Tokyo, Japan), located between four and ten centimeters above the antecubital fossa. A baseline measurement of brachial artery diameter was obtained, as well as a baseline measurement of the velocity of the arterial flow, by means of a pulsed Doppler signal of the vessel. After baseline measurements, a small-width blood pressure cuff was inflated on the most proximal portion of the forearm to occlusive pressure (300 mm Hg) for five minutes in order to induce hyperemia. The cuff was then deflated, and pulsed Doppler signals were recorded for 15 seconds. Images of the brachial artery were obtained after 60 seconds of cuff deflation. Vessel diameters were measured with ultrasonic calipers from the leading edge of the anterior wall to the leading edge of the posterior wall of the brachial artery at end diastole, incident with the R wave on the simultaneously recorded electrocardiogram. Changes in diameter were calculated as percentages of change relative to the baseline diameter. All images were recorded on Super VHS tape for later analysis. The studies were subsequently analyzed by two blinded observers; the mean values obtained from the two observers were used for analysis. The correlation in FMD between observers was 97.1% *P* < .00001, and the coefficient of variation was 8.59%. The carotid arteries were imaged with a Hewlett-Packard, Sonos 1500, Andover, Mass, USA ultrasound system with a lineal 7.5 MHz linear-array transducer. A depth of 4 cm was used. The examination included a thorough scan of the extracranial carotid arteries. The carotid IMT of the distal 1 cm of the far wall of the common carotid artery was performed using a semiautomated border-detection program by one single observer who was blinded to the clinical condition of the subject. The mean carotid IMT was calculated by averaging 3 measurements obtained at 3 scan planes (anterior, lateral, and posterior) from both the right and left common carotid arteries using the standard technique [26]. 

Before being included, and after full explanation of the purpose of the study, written consent was obtained from each subject. This study was carried out in adherence to the declaration of Helsinki and approved by the Ethics Committee of the Fundación Cardiovascular de Colombia.

### 2.1. Statistical Analyses

Student's *t*-test and Mann-Whitney tests were used to detect differences between groups according to the data distribution. To evaluate differences in vascular and inflammatory factors between groups and minimize possible interaction and confusion resulting from differences in basal parameters, we used an analysis of covariance (ANCOVA) that included the presence of CAD as a dependent variable and age, TG leves, and medication usage as covariates. The correlation between the plasma levels of vascular and inflammatory parameters was evaluated by Spearman correlation analysis and a simple linear regression. A *P*-value < .05 was considered statistically significant. Statistical analyses were performed with Stata Statistical Software: Release 10.0 SE. (College Station, Tex, Stata Corporation).

## 3. Results

Subjects' baseline characteristics are shown in Table 1. Excluding age and TG plasma concentration (patients +CAD were slightly older, and patients −CAD had higher TG plasma levels), no significant differences were observed in any anthropometric, metabolic, hemodynamic, or renal function parameters between the groups. Calcium channel blockers, beta-adrenergic blocking drugs, and salicylic acid were more commonly used by +CAD subjects (Table 2).

**Figure 1:**
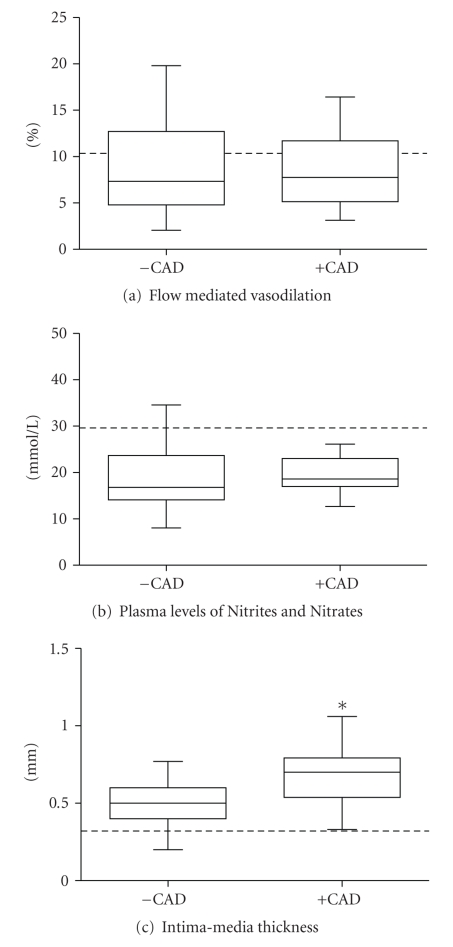
Box and whisker plots of carotid intima-media thickness, flow mediated dilation and plasma concentration of nitrites and nitrates in dyslipidemic subjects with (+CAD; *n* = 33) or without (−CAD; *n* = 69) symptomatic coronary artery disease (CAD). *P < .05 versus −CAD. Dotted lines represent the median of the values obtained from healthy subjects in the same laboratory.

**Figure 2:**
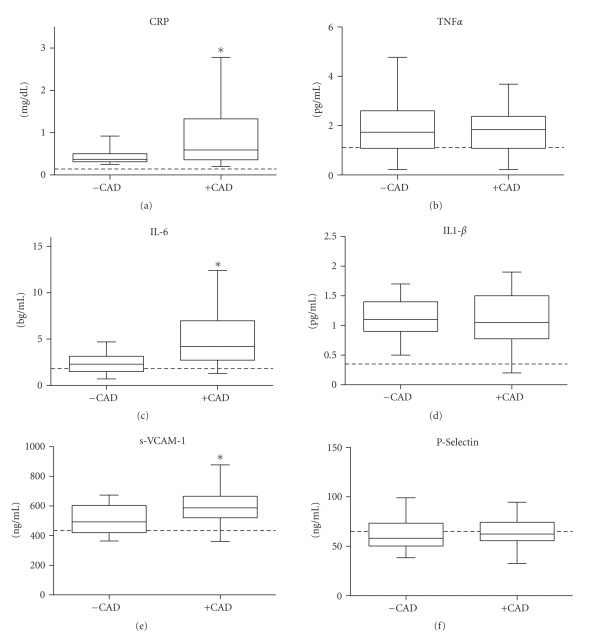
Box and whisker plots of plasma concentrations of C-reactive protein (CRP), interleukin 6 (IL-6), interleukin 1 beta (IL-1*β*), tumor necrosis factor alpha (TNF*α*), vascular adhesion molecule 1 (sVCAM-1) and P-Selectin in dyslipidemic subjects with (+CAD; *n* = 33) or without (−CAD; *n* = 69) symptomatic (CAD). *P < .05 versus −CAD. Dotted lines represent the median of the values obtained from healthy subjects in the same laboratory.

**Figure 3:**
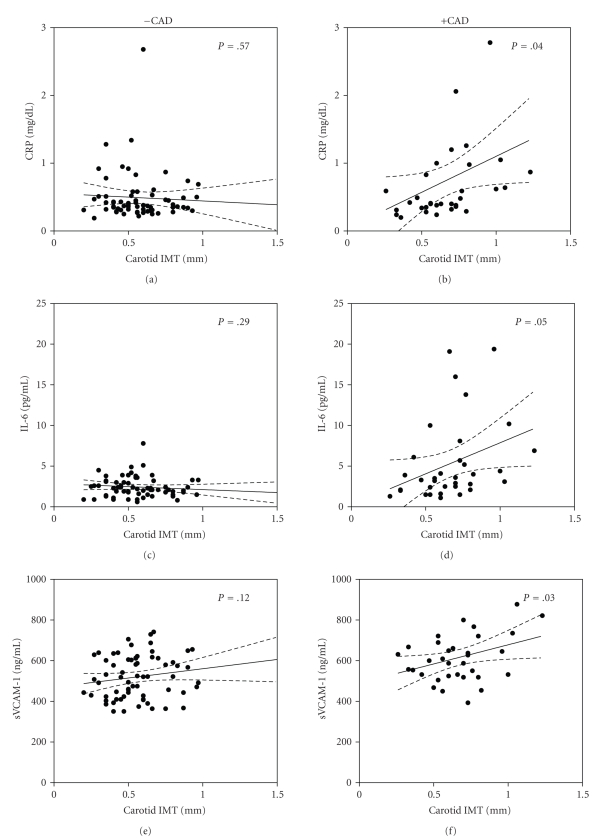
Scatter plots showing the correlation of intima-media thickness (IMT), and plasma levels of C-reactive protein (CRP; (a), (b)), interleukin 6 (IL-6; (c), (d)) and vascular adhesion molecule 1 (sVCAM-1; (e), (f)) in dyslipidemic subjects with (+CAD, *n* = 33) or without (−CAD, *n* = 69) symptomatic coronary artery disease. Solid lines represent the best-fit values for the linear regression, dashed lines represent the 95% confidence interval for linear regression, and *P*-values represent the probability for the slope of each regression being different to 0.

After adjusting for age, TG, and medication usage, no significant differences in FMD (ANCOVA *P* = .49) or plasma levels of nitrites (ANCOVA *P* = .54) were observed between patients with and without CAD (Figure 1). However, carotid IMT was significantly higher in subjects +CAD (ANCOVA *P* = .01) (Figure 1). Plasma levels of CRP, IL-6, and sVCAM-1 were higher (ANCOVA *P* < .05) in subjects +CAD when compared to subjects −CAD (Figure 2). There were no statistically significant differences in plasma concentrations of TNF*α*, IL-1*β*, and p-Selectin between the groups (Figure 2). Analyses of covariance showed no significant interaction between age and any of the endothelial or inflammatory parameters evaluated between the groups (*P* for interaction >.05). Table 3  shows values obtained from 25 healthy young Colombian subjects that were used in the study as reference values for normal conditions in our laboratory. Both groups of dyslipidemic patients (+CAD as well as −CAD) showed significantly lower values of FMD and levels of nitrites as well as higher carotid IMT and inflammatory markers than those from the reference group. Moreover, the carotid IMT was positively and significantly correlated to the plasma levels of CRP, IL-6, and sVCAM-1 in +CAD but not in −CAD subjects (Figure 3).

## 4. Discussion

The present study shows that dyslipidemic patients with a clinically documented history of CAD have higher concentrations of CRP, IL-6, and sVCAM-1 when compared to dyslipidemic patients without history of CAD. Interestingly, this elevation in certain inflammatory markers was not associated with any further impairment of endothelial function but was associated with a higher carotid IMT. Moreover, a positive correlation between the carotid IMT and plasma levels of certain inflammatory markers was present only in subjects +CAD. All together, these results suggest that there is an association between inflammation and the presence of a more severe stage of CAD.

In the previous years, a growing body of evidence has demonstrated the presence of endothelial dysfunction and increased concentrations of inflammatory markers in subjects with cardiovascular risk factors such as dyslipidemia [27, 28]. Furthermore, numerous reports support the importance of inflammatory markers as independent risk factors for cardiovascular events [15]. The results of the present study further support the concept that dyslipidemia is associated with endothelial function impairment and elevation of inflammatory markers [29, 30]. As expected, and independently of the presence of CAD, dyslipidemic patients had lower FMD and plasma nitrites as well as higher concentrations of CRP, IL-6, IL-1*β*, TNF*α*, and sVCAM-1 and carotid IMT than the reference healthy group.

This study also demonstrated that markers of endothelial dysfunction and inflammation were impaired in a differential manner depending on the existence of clinically diagnosed CAD. Endothelial dysfunction, evaluated by FMD and plasma levels of nitrates, was present to a comparable extent in both groups of patients, with or without CAD. Nevertheless, in those patients with a history of CAD, carotid IMT and some of the inflammatory markers measured (CRP, IL-6, and sVCAM-1) were higher than in patients without CAD. This indicates several aspects to be considered. One, that endothelial dysfunction and inflammatory markers do not represent the same degree of atherosclerosis progression (carotid IMT was higher in +CAD than in −CAD), nor the same degree of severity of cardiovascular disease. Two, that further elevation of inflammatory markers does not necessarily involve further reduction of FMD but does involve an augmentation of carotid IMT. Therefore, these findings suggest that once endothelial function is impaired by the presence of dyslipidemia, the presence of CAD is not associated with further impairment of endothelial function at least several months after the occurrence of the cardiovascular event. These results are consistent with a study recently published showing that, in older adults, the strongest predictors of cardiovascular events were age, sex, and blood pressure, and that FMD had a minimal effect on the evaluation of risk in this population [31, 32]. Moreover, the progression of atherosclerosis seems to be more severe in patients +CAD because they presented with a higher carotid IMT, which was also correlated with higher levels of CRP, IL-6, and sVCAM-1.

One of the interesting aspects of the study lies in the fact that none of the dyslipidemic subjects included received any lipid lowering medication, allowing us to evaluate the association between endothelial function, inflammatory markers, and clinical outcomes without the potential confusion caused by the well-described anti-inflammatory and pleiotropic effects of these kinds of medications [33–35]. Due to its transversal design, our results do not allow us to clarify whether the enhanced inflammatory process was a cause or consequence of the cardiovascular event. However, the differences in carotid IMT between groups suggest a role of inflammation in the progression of atherosclerosis and CAD. Moreover, the results strongly suggest that further elevation of certain inflammatory markers, such as CRP, IL-6, and sVCAM-1, could be considered as markers of severity of cardiovascular disease and consequently higher risk of developing a cardiovascular event in dyslipidemic subjects. Another interesting finding is the lower TG levels observed in +CAD patients compared to −CAD. Since there were no differences in any pharmacological intervention that could explain this finding, we believe that it could be attributable to the fact that +CAD subjects usually follow dietary recommendations more strictly that −CAD patients. Given that age is a factor that can affect the presence of inflammatory markers and endothelial function, one limitation of this study is the absence of a control group of age-matched patients with “normal" cholesterol levels with and without atherosclerosis. Therefore, the obtained results can only be extrapolated to dyslipidemic subjects.

In summary, the present study indicates that in dyslipidemic subjects further elevation of certain inflammatory markers is associated with increased carotid IMT and history of CAD, but not with further endothelial dysfunction. The results also suggest that inflammatory markers and carotid IMT, associated with the presence of clinically diagnosed CAD are partially independent of the lipid profile and the degree of endothelial dysfunction.

## Figures and Tables

**Table 1:** Anthropometrical, metabolic and biochemical characteristics of subjects with (+CAD) and without (−CAD) coronary artery disease.

	−CAD (*n* = 69)	+CAD (*n* = 33)
	Median (IQR)	Median (IQR)
Age (years)	50.7 (44.3–57.7)	58.4 (52.5–66.6)*
BMI (Kg/m^2^)	25.6 (24.3–27.3)	25.1 (22.5–26.9)
Waist Circumference (cm)	91.2 (84–96)	89.5 (82.2–97.6)
Hip Circumference (cm)	92.5 (86.7–98)	91.5 (86–98)
W/H	0.98 (0.96–1.01)	0.98 (0.95–1.02)
SBP (mmHg)	120 (110–131)	125.5 (112.5–140)
DBP (mmHg)	79 (70–87.5)	79.5 (70–86.5)
Alanine aminotranferease (UI/L)	28 (22–36)	24 (18–32)
Aspartate aminotransferase (UI/L)	22 (19–25)	22 (17–25)
Glucose (mmol/L)	5.2 (4.9–5.5)	5.2 (5.01–5.61)
Creatinine (*μ*mol/L)	96.3 (87.5–104.3)	97.6 (85.7–105.2)
TC (mmol/L)	6.5 (5.98–7.18)	6.04 (5.53–6.89)
HDLc (mmol/L)	0.93 (0.8–1.07)	0.90 (0.77–1.07)
LDLc (mmol/L)	4.45 (3.77–5.01)	4.49 (3.93–5.06)
TG (mmol/L)	2.14 (1.68–2.86)	1.62 (1.21–2.40)*

BMI: Body mass index; W/H: Waist to hip ratio; SBP: Systolic blood pressure; DBP: Diastolic blood pressure; Glucose: Fasting plasma glucose; Creatinine: Creatinine plasma levels; TC: Total cholesterol plasma concentration; HDLc: High density lipoprotein plasma concentration; LDLc: Low density lipoprotein plasma concentration; TG: Triglyceride plasma concentration. IQR: Interquartile range. *P < .05 versus −CAD.

**Table 2:** Permanent medication usage in dyslipidemic subjects with (+CAD) and without (−CAD) coronary artery disease.

	−CAD (*n* = 69)	+CAD (*n* = 34)
Medication	Permanent usage %	Permanent usage %
ACEIs	17.4	32.4
Aspirin	21.7	73.5*
NSAI	26.1	17.6
CCA	7.2	26.5*
Beta blockers	4.3	61.8*
Digoxin	1.4	2.9
Furosemide	2.9	0.0
Hydrochlorothiazide	2.9	8.8
clopidogrel	0.0	2.9

ACEIs: Angiotensin-converting enzyme inhibitors, NSAI: Nonsteroidal anti-inflammatory agents, CCA: Calcium channel antagonists, *: *P* < .005 versus –EC.

**Table 3:** Reference values obtained from 25 healthy young Colombian subjects.

Variable	Median (IQR)
FMD (%)	10.74 (7.9–13.5)
Nitrites (mmol/L)	29.8 (22.8–32.9)
IMT (mm)	0.31 (0.23–0.42)
CRP (mg/dL)	0.15 (0.12–0.17)
IL-6 (pg/mL)	1.21 (0.72–1.92)
IL-1*β* (pg/mL)	0.34 (0.23–0.8)
TNF*α* (pg/mL)	1.06 (0.53–1.59)
sVCAM-1 (ng/mL)	425.5 (374–584)
P-Selectin (ng/mL)	66.8 (52–85)

FMD: Flow mediated dilation, IMT: Carotid intima-media thickness, CRP: plasma concentrations of C-reactive protein, IL-6: Interleukin 6, IL-1*β*: Interleukin 1beta, TNF*α*: Tumor necrosis factor alpha, sVCAM-1: Vascular adhesion molecule 1; IQR: Interquartile range.
